# Partnering with patients in translational oncology research: ethical approach

**DOI:** 10.1186/s12967-017-1177-9

**Published:** 2017-04-08

**Authors:** Marie-France Mamzer, Nathalie Duchange, Darquy Sylviane, Patrice Marvanne, Claude Rambaud, Giovanna Marsico, Catherine Cerisey, Florian Scotté, Anita Burgun, Cécile Badoual, Pierre Laurent-Puig, Christian Hervé

**Affiliations:** 1 Laboratoire d’Ethique Médicale et Médecine Légale EA4569, Faculté de Médecine, Université Paris Descartes, 45 rue des Saints-Pères, 75006 Paris, France; 2 Unité fonctionnelle d’éthique et médecine légale, Hôpital Necker-Enfants malades, Assistance publique-Hôpitaux de Paris, 75015 Paris, France; 3 Independent patient representative, Paris, France; 4 Collectif Interassociatif Sur la Santé (CISS), 75007 Paris, France; 5 Cancercontribution.fr, Paris, France; 6 Patients & Web, Paris, France; 7 Soins de support, Service de cancérologie, Hôpital Européen Georges Pompidou, Assistance publique-Hôpitaux de Paris, 75015 Paris, France; 8 Département d’informatique médicale, de biostatistique et de santé publique, Hôpital Européen Georges Pompidou, Assistance publique-Hôpitaux de Paris, 75015 Paris, France; 9 UMR-S 1138, Centre de recherche des Cordeliers, 75006 Paris, France; 10 Faculté de médecine Paris Descartes, Sorbonne universités, Paris, France; 11 Centre de Ressources biologiques, Service d’anatomo-pathologie, Hôpital Européen Georges Pompidou, Assistance publique-Hôpitaux de Paris, 75015 Paris, France; 12 Inserm UMR-S 1147, Université Paris Descartes, 75006 Paris, France; 13 Service de Biochimie Pharmacogénétique et Oncologie Moléculaire, Hôpital Européen Georges Pompidou, Assistance publique-Hôpitaux de Paris, 75015 Paris, France

**Keywords:** Medical ethics, Personalized medicine, Patient-centered approach, Translational research, Translational ethics

## Abstract

**Background:**

The research program CARPEM (cancer research and personalized medicine) brings together the expertise of researchers and hospital-based oncologists to develop translational research in the context of personalized or “precision” medicine for cancer. There is recognition that patient involvement can help to take into account their needs and priorities in the development of this emerging practice but there is currently no consensus about how this can be achieved. In this study, we developed an empirical ethical research action aiming to improve patient representatives’ involvement in the development of the translational research program together with health professionals. The aim is to promote common understanding and sharing of knowledge between all parties and to establish a long-term partnership integrating patient’s expectations.

**Methods:**

Two distinct committees were settled in CARPEM: an “Expert Committee”, gathering healthcare and research professionals, and a “Patient Committee”, gathering patients and patient representatives. A multidisciplinary team trained in medical ethics research ensured communication between the two committees as well as analysis of discussions, minutes and outputs from all stakeholders.

**Results:**

The results highlight the efficiency of the transfer of knowledge between interested parties. Patient representatives and professionals were able to identify new ethical challenges and co-elaborate new procedures to gather information and consent forms for adapting to practices and recommendations developed during the process. Moreover, included patient representatives became full partners and participated in the transfer of knowledge to the public via conferences and publications.

**Conclusions:**

Empirical ethical research based on a patient-centered approach could help in establishing a fair model for coordination and support actions during cancer research, striking a balance between the regulatory framework, researcher needs and patient expectations. Our approach addresses the concept of translational ethics as a way to handle the main remaining gap between combining care and research activities in the medical pathway and the existing framework.

## Background

The underlying promise of personalized/precision medicine is to adapt diagnosis, treatment and prevention to patients according to their genetic and molecular profile, lifestyle and environments. This concept has been strengthened by many therapeutic and diagnostic advancements in oncology [1–5]. The challenge is both to accelerate the translation of biomedical research to therapeutic application [6]—from bench to bedside and to use patient samples and data and samples for research purposes in order to bring relevant clinical needs into the laboratory research environment—from bedside to bench to bedside [7].

Yet, personalized medicine is not a continuum; indeed, it results from a complex and still-emerging approach to medicine that underlies ongoing translational research (TR) programs. TR relies in part on non-interventional research, and the emergence of next-generation sequencing technologies for personalized medicine blurs the traditional dichotomy between research and clinical practice. TR is driven by the outgrowth of infrastructures gathering health data and samples on a large scale. It relies on the combination of clinical, genomic, biological data as well as the availability of biological material in resource centers. It also relies on the de-compartmentalization of data gathered at national, pan-national or disease levels [8, 9]. A growing body of literature highlights the need to rethink the current bioethical and regulatory frameworks [7, 10–14] and as Nicol et al. [13] postulate, to reformulate in this context the classical tensions between community welfare and individual liberty, risk and benefit, and autonomy and paternalism. Indeed, on one hand, data and samples may be re-used in future unknown research and on the other, genetic or genomic analysis may result in a diagnostic or therapeutic application and in proposals to participate in a clinical trial of precision medicine [15–17].

CARPEM (cancer research for personalized medicine) is a French consortium certified in 2013 which develops translational research in the context of personalized or “precision” medicine for cancer. CARPEM’s goals are to perform innovative and efficient translational research, stimulate data sharing and collaboration among researchers, and accelerate the translation of biomedical research to therapeutic application to benefit patients. To achieve these goals, CARPEM developed three integrated research programs. Two are scientific TR ones, dealing with complementary approaches of personalized medicine (genomic and cellular). The third is an empirical-ethical research program exploring the feasibility of building, together with patients and patient representatives, some ethical guidelines to bridge the persistent although inappropriate division between research ethics and medical ethics.

In this paper, we describe the establishment, methodology and first results of the empirical-ethical research program of CARPEM. We describe how the mediation between two independent ethics committees by a multidisciplinary team of researchers specialized in medical ethics allowed for (1) the transfer of knowledge between parties ensuring a path to transparency for the public and (2) the identification and handling of ethical issues jointly by professionals and patient representatives. Finally, we discuss how we are thinking the translational ethics approach to establish a fair model for coordination and support actions in TR, striking a balance between the French legal framework, researcher needs and patient expectations. With such an empirical approach, we aim to surpass the limitations of abstract ethical reasoning and address “real life” ethical issues in the context of a concrete personalized medicine program, CARPEM.

## Methods

### Analysis of practices

To describe current practices in CARPEM and organize the sharing of knowledge between all stakeholders, we created two distinct multidisciplinary committees: an “Expert Committee”, gathering healthcare and research professionals, and a “Patient Committee”, gathering patients and patient representatives. The team of the Laboratory of Medical Ethics (LEM) of University Paris Descartes provided mediation between both committees. The LEM team is in charge of the agenda and minutes of all meetings and consists of specialists in medical ethics, research ethics, law, anthropology and linguistics. Participants of each committee validated the minutes.

### Expert Committee

The Expert Committee functioned as a “chat room” between practitioners and researchers participating in the medical and scientific aspects of the CARPEM program. The committee met monthly from February 2013 to September 2014 and quarterly thereafter until December 2015. The committee was involved in collecting and disseminating information about the aims of CARPEM. Healthcare and research professionals shared their experiences, difficulties encountered and expectations. Each meeting was dedicated to a speciality involved in personalized medicine—oncology, biology, biomedical informatics, cancer genetics, radiology, psychology, dietetic and primary care medicine—which ensures the continuity of care between the hospital and home/rehabilitation unit. The LEM team presented reports of these meetings to the Patient Committee.

### Patient Committee

The Patient Committee was set up as (1) a forum for exchange and communication with patients; (2) an advisory committee that gathered patient expectations, fears or criticisms about personalized medicine; and (3) a body to provide recommendations and validate the deliverables from the Expert Committee. The Patient Committee consisted of six patients participating in the CARPEM research program, two representatives from patient associations and one representative of the public, together with the LEM team. Meetings occurred every 2 months from February 2013 to December 2015. The LEM team presented the discussions from the Expert Committee, collected the views expressed in the Patient Committee, produced minutes for each meeting and reported them to the Expert Committee.

### Transfer of knowledge to the public

CARPEM is committed to informing and educating patients and the public via its website and by producing educational videos [18]. Social media channels (YouTube, LinkedIn and Twitter) are also used. All videos are produced in French with French and English subtitles.

The subprogram ethics and Cancer organized an agenda for disseminating knowledge and guidelines through international workshops that are open to the public (in collaboration with the Société Française et Francophone d’Ethique Médicale and the International Institute of Research in Ethics and Biomedicine). Topics were chosen to ensure audience interest in precision medicine and patient-centered care.

## Results

### Building a common knowledge base for co-construction of CARPEM translational research practices

**Fig. 1:**
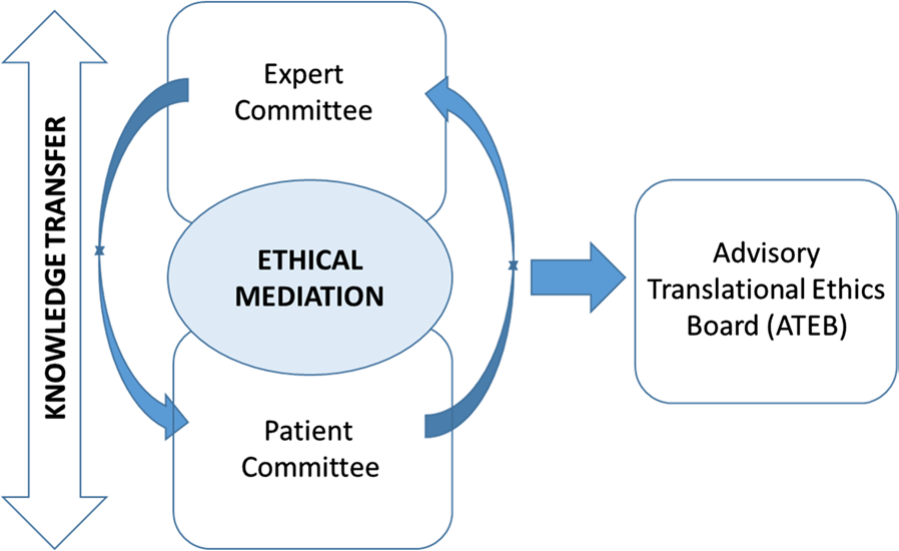
Description of the ethics committees in CARPEM. The Expert Committee and the Patient Committee operated for 2 years before being merged into the Advisory Translational Ethics Board (ATEB)

The Expert and Patient committees had numerous exchanges for establishing fair information processes and building proper practices (Fig. 1). With the emergence of personalized medicine, healthcare professionals in the Expert Committee recognized the need to consider all the implications of this medicine, particularly in terms of responsibilities and derivative practices. They acknowledged that the development of personalized medicine would involve profound changes in practices. They felt that the Expert Committee, as a unique place to meet, balanced with the current “silo organisation” of their own practices and gave them the opportunity to share their thoughts on their expectations and concrete difficulties. The Expert Committee meetings were also a forum for professionals to hear about patient expectations, fears and claims as transmitted by the LEM team after each Patient Committee meeting. This organisation in two committees has resulted in a form of research ethics consultation service helping professional teams to identify ways to address the ethical policy and social challenges born in TR [19, 20]. In our model, patients’ representatives are closely embedded.

After a few meetings of the Patient Committee, patients criticized the term “personalized medicine”, the most common term used at the time the project started. They felt that this label was confusing because it suggested patient-centered medicine, but they came to realize that it refers to a molecular/genomic approach that aims for an overall set of objectives including preventive, predictive and precision medicine. Moreover, patients realised that precision medicine implied the process of TR.

During their exchanges with the LEM members, all participants of the two committees could upgrade their knowledge about the ethical and legal norms for both practices of TR: research and healthcare. They progressively understood why and how personalized medicine (or most appropriately, precision medicine) was erasing boundaries between the clinic, laboratory and healthcare industry.

### Addressing complexity

When the two committees first met, CARPEM was creating its own TR platform by transforming existing clinical biorepositories into a unique research-oriented one integrating large collections of patient samples together with clinical, pathologic and outcome data stored in a unique data warehouse [15, 21, 22]. The previous patient information process was inappropriate because many human samples were collected during the care pathway without gathering any informed consent for research purposes or were accompanied by an obsolete care one. This lack of individual informed consent represented an insurmountable obstacle to the re-use of clinical samples for TR, both for ethical and legal reasons. However, the overall discussions highlighted the complexity of the information to deliver. One element of complexity patients identified was that at the global level, precision medicine is usually described as a continuum between research and care, whereas at the individual level, discontinuity might be the rule. Thus, patients understood that a patient who consents to giving samples and data for precision medicine purposes has no assurance of therapeutic individual benefit nor any kind of reciprocity. Moreover, the research results could expose the patient to incidental findings. Professionals are indeed worried about this eventuality. Furthermore, all stakeholders, professionals and patients, agreed that the complexity is due in part to the multiplicity of actors involved, the technical complexity of data or sample gathering, and the uncertain temporality of data or sample re-use. They urged the need to update and standardize patient information and informed consent forms, even though building fair informed consent processes seemed a challenge in the context of TR and precision medicine.

### General recommendations from the Patient Committee

Patients and their representatives in CARPEM highlighted from the outset the need for their involvement in the design of “precision medicine”. They cited specific concerns.

First, they wanted information that would help them make informed decisions, in particular concerning the use and re-use of routine care data and samples. They also recommended clarifying the process of information and consent in terms of organisation and professional responsibilities.

Second, they were worried about how a number of sensitive elements would be explained to patients: patients’ possible ineligibility to receive innovative treatments without feeling abandoned by medicine, the possible discontinuation of treatment due to the non-sustainability of some therapeutic targets, and finally, the coordination of their medical care within the community–hospital interface, not yet established.

Accordingly, the Patient Committee developed the following four recommendations [23]: (1) the patient and family should be properly informed of the freedom to accept or decline precision medicine or TR participation without judgment of any kind (a fair and balanced information should cover both the treatment options and the foreseeable consequences of the results); (2) privacy should be ensured; (3) data and/or samples collected in CARPEM should be used to precision medicine goals; and (4) patients and groups representing the public should be included in all decisions and processes.

### Contribution of the Patient Committee to producing an information leaflet on searching for genetic alterations in cancer

The Patient Committee contributed to the elaboration of a leaflet describing the process for searching for genetic alterations in tumor by sequencing in view of a potential targeted therapy to be delivered during a consultation in oncogenetics. The committee considered important a number of points that would better inform patients, which allowed for establishing a framework for guidance: (1) the leaflet should use “you” as much as possible and information should be contextualized to the patient’s situation; (2) it should briefly describe the method of analysis comparing the genetic material found in tumor and blood samples; (3) it should specify the delay in communicating results; (4) it should address the risks of incidental findings, and the option to be informed or not should be proposed and revised over time; (5) it should give details about samples and data such as storage place, length of conservation, and potential re-use for research purposes; and (6) a group of patients or their representatives should carefully review all leaflets.

The leaflet produced closely followed this guidance. Particularly with regard to unintended outcomes, the discussions resulted in the following organization: (1) patients are informed individually about the possibility of incidental findings by clinician researchers, both verbally and in the information leaflet and (2) the consent form allows the patient to express his or her preferences as to whether or not to be informed of the outcome. If the patient chooses to know the results of an incidental finding whose clinical relevance is established, this is done during an oncogenetic consultation. A diagnostic test is then proposed to the patient in order to validate the result of sequencing technologies.

### Contribution of the Patient Committee to a consent form addressing the re-use of data and samples

Professionals reported the difficulty in reusing a large number of data and samples collected during cancer care for research aims despite their potential use for research purposes [24, 25]. This difficulty is due to the absence of prior patient information and the lack of collecting appropriate consent for this. To handle this issue, existing consent forms were collected. Their analysis revealed their diversity, their lack of information on new technologies, and the need for legal and regulatory updates [26].

There was an urgent demand from professionals to have a generic and shared consent form that could be adapted to different situations. A framework for a consent form was worked out to support prospective research projects (interventional research) and another to support storage and retrospective research on samples and data (non-interventional research).

The CARPEM Management Team implemented a new informed consent form for all cancer patients in hospitals to encompass the collection of tumor samples and data for research. The Patient Committee discussed the informed consent form before its approval by a French legal ethics research committee. The form allows for conserving data and samples for future research in the field of cancer. The collection of tumor samples with use of the new consent form is ongoing. To better organize and improve the systematic collection and processing of patient consent, two biomedical engineers were recruited by the CARPEM in April 2015. They are working with the local clinical teams to improve the circulation of patient information and the collection of consent.

### Recognition of patients as peers in CARPEM

After 3 years of existence (2013–2015), the Expert and Patient committees were merged into a single committee, the Advisory Translational Ethics Board (ATEB). The ATEB functions as an advisory committee overseeing the ethical and cultural dimensions of all CARPEM projects. It deals with any ethical issues that might emerge during the process of TR. It meets once per quarter. One of the first objectives of the ATEB was to work with both healthcare professionals and patients to build useful tools for a better understanding of a “fair exchange of information in order to help patients make informed decisions”. As a result of the patient engagement in CARPEM projects, a patient representative was included in the CARPEM Steering Committee.

Because of their active participation in CARPEM, patients and their representatives acquired the knowledge and skills to animate the public debate on precision medicine. Their intervention as speakers and moderators to scholarly conferences is key to educating the public about precision medicine.

### Transfer of knowledge to the public

CARPEM has produced 10 short videos (from 1 to 5 min) containing interviews with CARPEM researchers, and physicians. The videos explain precision medicine and how it can change clinical practice. Two videos are dedicated to ethical issues, one involving an interview with a LEM coordinator and the other patients themselves. They are available on the CARPEM website (http://www.carpem.fr).

Five 1-day workshops dedicated to ethics occurred between March 2013 and December 2015. They addressed “Complex computerized systems in health”, “New paradigms of personalized or precision medicine”, “Platforms for access to care and research”, “Social disparity and health” and “Medical confidentiality, the right of oblivion and personalized medicine in cancer”. Conference proceedings were produced for each meeting and were published in French [27, 28].

## Discussion

Translational research is a complex process that encompasses traditional biomedical research. The latter deals with experimentation on human subjects and originates from the ethics codes of the mid-to-late 1900s [10]. These codes aim to protect research participants (patients and volunteers) enrolled in interventional and physically risky research. The CARPEM program is dedicated to precision medicine and TR in oncology, benefiting patients from recent discoveries and re-formulating research questions from the needs identified by the clinic. It integrates ethical expertise in its governance and develops ongoing empirical-ethical research to identify, support and fuel the debate about ethical issues encountered in real-life practice. To this end, patients were included during the entire process, giving them the “floor” to express their vision and expectations and engage them in constructing an appropriate normative/ethical framework.

This organization in two committees has resulted in a form of research ethics consultation service helping professional teams to identify ways to address the ethical policy and social challenges born in TR [19, 20]. In our model, patients’ representatives are closely embedded.

A first aim of the ethics program was to facilitate knowledge transfer among patients, their representatives, and the different kinds of healthcare professionals so as to build a common language and common understanding. This aim was realized by the interaction of two ethics committees, the Expert Committee and Patient Committee, that met for more than 2 years before being merged. This is key to enabling effective patient involvement not only as active participants but also as real partners whose inputs are valuable throughout the process [29]. Such a transfer of knowledge was essential to ensure transparency and to allow patients and society to accept these new practices [13].

A second—and still ongoing—objective was the identification of the ethical issues at stake. One of the main issues arising from the committee discussions and highlighted by patients was the primary dual intentionality of TR, bridging care and research pathways. Recently, Hostiuc et al. [7] divided TR into a succession of six operating phases, starting with fundamental research and ending with the implementation of social policies, as a result of the whole translation process. Indeed, “translation” usually refers to how biomedical science should produce a return of investment by bridging the gap between the amount of knowledge produced and the expected benefit, the “bench-to-bedside” approach [30, 31]. In CARPEM, particular attention in the translation movement was paid to the bedside-to bench approach because TR rests on translational researchers who are also physicians at the interface between the clinic and research. Indeed, a very important part of the translational process consists of harvesting and storing patient samples and data collected for both diagnosis or care and research. This process is insufficient as such and has to be accompanied by the questions posed by clinician researchers. This is indeed an effective mean to transfer the clinical problems that need to be addressed into the laboratory research environment.

Patients became aware of the dual translation movement and accepted this. They provided a number of recommendations related to the information and consent process for the use of data or samples for research, thereby reaffirming their attachment to the principle of autonomy. Finally, they participated in elaborating two kinds of information leaflets, one for searching for genetic alterations in tumors with potential medical consequence for the patient and one concerning the future use of data or samples. This process agrees with recognizing that the legal and ethical frameworks in precision medicine and that TR must be built with a patient-centered approach, taking into account the individual concerns and expectations of patients [7, 13, 14, 32, 33].

This process has been a first step in harmonizing the ethical procedures in CARPEM by pragmatic consensual solutions consistent with the existing legal and ethical framework and convenient to all parties. Thus, the ethical process was shaped in the research program by integrating these approved measures as structural constraints while respecting the scientific objectives. Indeed, a recommendation was that the modernization of the current regulatory landscape for precision medicine should involve mobilizing as much as possible the existing regulatory instruments and ethical principles [13].

However, our results revealed ethical issues that need to be worked out at the international level when no pragmatic and consensual solutions compatible within the current framework can be found. Consent models for TR represents one of the core ethical, legal, and social issues in bioethics. For example, a concern is that the broad consent process adopted by many studies as a practical solution for unforeseen secondary research aims may actually reduce the trust between participants and researchers [9, 24]. Indeed, the Patient Committee argued for a specific and “related conditions” consent [9] for re-use of data and samples limited to the goal of precision medicine. The committee recommended, for any other purpose, a labelled ethical research committee statute on the need to re-contact patients, taking into account the terms of the initial consent form.

Our results are consistent with the opinion of some authors who stressed the need for a specific set of bioethical guidelines for TR and proposed the concept of “translational ethics” [7, 10–12]. The authors emphasize that this set should be developed according to the steps or phases identified in the process and to the entire translational research process. As early as 2000, Kagarise and Sheldon introduced “translational ethics’ in an attempt to account for the intertwining of research and clinical practices in TR [10]. The authors recommended a model of procedural ethics based on autonomy and informed consent. They stressed the difficulty in providing such informed consent in unprecedented situations inherent in modern practices and advocated for an initial and ongoing educational dialogue between patients and physicians that would require new competencies for physicians [10]. Their reasoning was developed from a moral and professional responsibility perspective.

Our approach goes further: first, it embeds the patient perspective from the beginning in a concrete academic and institutional TR program, and second, it addresses the acceptability of the translational research process itself. In 2009, Cribb [12], discussing the concept of translational ethics under the perspective of the theory–practice gap in medical ethics, highlighted the interest of reflecting on the bridge linking ethical scholarship to practice. He argued that translational ethics should refer to the “what” and “how” dimensions. The “what” implies reflection on the meaning of practices, step by step and globally, whereas the “how” considers their modalities of implementation. For Cribb, medical ethics translation cannot work on the “how” dimension in isolation but needs to account for whether the resulting actions are ethically justifiable. By doing this, Cribb stresses the risk of “ethics dumping” and blind transposition of ethical regulations that are not adapted to current practices. To avoid this risk and to facilitate the acceptance of new practices by the public at both the individual and global levels, the patient-centered approach is key [33]. We applied this approach in CARPEM and demonstrated its relevance.

## Conclusions

The current debate surrounding precision medicine and translational research focuses merely on the need for a new international regulation framework, but a number of unexplored ethical issues need to be addressed in the “real life” context. A way to progress is to open the dialog between professionals, patients and society. One hypothesis in CARPEM was that empirical ethical research based on a patient-centered approach should help establish a fair model for coordination and support actions for translational cancer research. This involves striking a balance between the regulatory framework, researcher needs and patient expectations. Our results demonstrate the relevance of including patients and representatives to achieve this primary goal and to ensure transparency and trust. The recognition of patients as peers by professionals led to their concrete participation in framing precision medicine practices and to their participation, as ambassadors, in diffusing knowledge to the public. In the end, they were integrated as co-deciders in the steering committee.

## Abbreviations

CARPEM: cancer research and personalized medicine
TR: translational research
