# Erratum to: Partnering with patients in translational oncology research: ethical approach

**DOI:** 10.1186/s12967-017-1181-0

**Published:** 2017-04-23

**Authors:** Marie-France Mamzer, Nathalie Duchange, Sylviane Darquy, Patrice Marvanne, Claude Rambaud, Giovanna Marsico, Catherine Cerisey, Florian Scotté, Anita Burgun, Cécile Badoual, Pierre Laurent-Puig, Christian Hervé

**Affiliations:** 1 Laboratoire d’Ethique Médicale et Médecine Légale EA4569, Faculté de Médecine, Université Paris Descartes, 45 rue des Saints-Pères, 75006 Paris, France; 2 Unité fonctionnelle d’éthique et médecine légale, Hôpital Necker-Enfants malades, Assistance publique-Hôpitaux de Paris, 75015 Paris, France; 3 Independent patient representative, Paris, France; 4 Collectif Interassociatif Sur la Santé (CISS), 75007 Paris, France; 5 Cancercontribution.fr, Paris, France; 6 Patients & Web, Paris, France; 7 Soins de support, Service de cancérologie, Hôpital Européen Georges Pompidou, Assistance publique-Hôpitaux de Paris, 75015 Paris, France; 8 Département d’informatique médicale, de biostatistique et de santé publique, Hôpital Européen Georges Pompidou, Assistance publique-Hôpitaux de Paris, 75015 Paris, France; 9 UMR-S 1138, Centre de recherche des Cordeliers, 75006 Paris, France; 10 Faculté de médecine Paris Descartes, Sorbonne universités, Paris, France; 11 Centre de Ressources biologiques, Service d’anatomo-pathologie, Hôpital Européen Georges Pompidou, Assistance publique-Hôpitaux de Paris, 75015 Paris, France; 12 Inserm UMR-S 1147, Université Paris Descartes, 75006 Paris, France; 13 Service de Biochimie Pharmacogénétique et Oncologie Moléculaire, Hôpital Européen Georges Pompidou, Assistance publique-Hôpitaux de Paris, 75015 Paris, France

## Erratum to: J Transl Med (2017) 15:74 DOI: 10.1186/s12967-017-1177-9

In the original version of this article [1], published on 8 April 2017, the name of author ‘Sylviane Darquy’ was wrongly displayed.

Originally the author name has been published as:

- Darquy Sylviane

The correct author name is:

- Sylviane Darquy

The original publication of this article has been corrected.
